# Non-Immortalized Human Tenocyte Cultures as a Vehicle for Understanding Cellular Aspects to Tendinopathy.

**Published:** 2011-10-17

**Authors:** L. Yao, C.S. Bestwick, L.A. Bestwick, R.M Aspden, N. Maffulli

**Affiliations:** (1) Department of Orthopaedic Surgery, University of Aberdeen, Foresterhill, Aberdeen AB25 2ZD, United Kingdom.; (2) Cellular Integrity Programme, Rowett Research Institute, Greenburn Road, Bucksburn, Aberdeen, United Kingdom, AB21 9SB.; (3) Centre for Sport and Exercise Medicine, Barts and the London School of Barts and The London School of Medicine and Dentistry, Mile End Hospital, 275 Bancroft Road, London E1 4DG, England

**Keywords:** Tendinopathy, Cell Cultures, Exercise, Oxidative Stress, Tenocyte cultures

## Abstract

The biochemical mechanisms underlying tendinopathy are obscure. We briefly describe preliminary observations of human tenocyte behaviour in culture as a vehicle for determining the role of reactive oxygen in tendon pathology.

Tendons transfer the force from muscular contraction to bone. The term ‘tendinopathy’ includes the pathologies in and around tendons (1, 2). In most instances, sports-related tendinopathies result from a dysfunctional repair response (1, 2). This histopathological appearance has been termed “tendinosis”, although the degenerative implication of this label is only partially correct, as the histopathological picture is of a failed, haphazard healing response (3). Overuse tendinopathies show no evidence of “tendonitis” (i.e. of a local inflammatory reaction), providing a histopathological explanation for the chronicity of symptoms that often occur in athletes with tendinopathies.

Tendinopathy involves both the collagen matrix and the specialised tendon fibroblasts, the tenocytes. Normally, collagen fibres in tendons are tightly bundled in a parallel fashion, but tendinopathic samples show unequal and irregular crimping, loosening and increased waviness of collagen fibres, with an increase in Type III (reparative) collagen (4, 5). In tendinopathic tendons, tenocytes are abnormally plentiful in some areas, and have rounded nuclei and ultrastructural evidence of increased production of proteoglycan and protein which gives them a chondroid appearance. Other areas may contain fewer tenocytes than normal with small, pyknotic nuclei (4). Rarely, there is infiltration of lymphocytes and macrophage type cells, which may be part of a healing process (4). Although classically tendinopathy was thought to be associated with hypovascularity, a characteristic feature of tendinopathic tendons is proliferation of capillaries and arterioles, with degeneration of tenocytes and collagen fibres, and subsequent increase in noncollagenous matrix (6, 7).

Two aetiopathogenetic hypotheses have been propounded for the occurrence of tendon rupture within tendinopathy. First, that injury *per se* may only be manifest after considerable underlying tendon damage, as described above. Second, that injury occurs by more sudden excessive mechanical forces without a requirement for degeneration (8–11). There may be overlap between these two hypotheses (12).

Healing of ruptured tendons depends on the intrinsic potential of the tenocytes to respond to the stimulus induced by the injury to the surrounding tissue matrix (13). This will be manifest within a complex series of cellular responses possibly encompassing apoptosis (programmed cell death), chemotaxis, proliferation, and differentiation (5, 14–16). However, the relative occurrence and importance of these events, the balance of which will be crucial in determining the effectiveness of repair and any prevalence to repetitive damage, remains obscure. In addition, the mechanism of failed healing response which may predispose the tendon to mechanical damage may also be superimposed on other, not yet clarified, processes.

A molecular link between the apparently disparate events of overuse tendon injuries and the subsequent orchestration of effective healing may well be the control of the production and persistence of a variety of molecules within both the intra and extracellular tendinous environment (17).

## Cellular responses to reactive oxygen

Radical and non-radical but reactive species oxygen species (ROS) include the superoxide anion (O_2^.−^_), hydrogen peroxide (H_2_O_2_), hydroxyl radical (HO^.^), singlet oxygen (O_2_^1^), peroxyl radicals (RO_2^.^_), and the interrelated reactive nitrogen species, peroxynitrite.

ROS may induce cellular/tissue damage via lipid peroxidation, DNA damage and protein modification (18–21). The participation of H_2_O_2_ in the iron/copper catalysed Fenton and Haber-Weiss reactions to form highly reactive HO. probably accounts for much of the *in vivo* toxicity associated with excessive O_2_^.−^/H_2_O_2_ production (19–21). Conditions in which excessive ROS generation are thought to be of direct importance include, but are no way confined to, tumourigenesis, coronary heart disease, autoimmune disease (18, 20). ROS are also implicated in overuse exercise- related damage in muscle (9), and may impair fracture healing in bone (22).

ROS levels are determined by the balance between their generation and antioxidant defence mechanisms. Antioxidant defences and regulators include endogenous enzymes (e.g. superoxide dismutase, catalase, glutathione reductase, glutathione peroxidase), iron/ copper chelators (e.g. ferritin, metallothioneins) endogenous low molecular weight compounds (e.g. glutathione, thioredoxin, urate). Other compounds as well as scavenging ROS are also able to repair ROS mediated damage and exert direct effects on redox mediated gene activation/repression (e.g. thioredoxin) (18, 23, 24). In addition, various dietary compounds such as vitamin C and E, exhibit antioxidant activity, and may complement endogenous antioxidant defences (18–20).

The association with direct pathological damage has to a certain extent obscured observations that changes in ROS type and concentration may exert more subtle effects on cell metabolism and development. ROS act as intra- and possibly inter-cellular signal molecules influencing signal transduction pathways and gene expression, and have been implicated in the processes of cell proliferation (25), differentiation (26), and stress adaptation (9, 19). ‘Higher’ levels of ROS may induce the demise of the cell either via direct damage or through the activation of and/or participation in ‘active’ cell death mechanisms (27–29).

Thus the effects of altered antioxidant/prooxidant activity are manifest in a diversity of cellular responses. We suggest that it is the assessment of altered cellular function and viability defines whether ‘end point’ labels such as ‘damaging’ and ‘stressed’ can really be applied to any such changes.

### Tendons and reactive oxygen

Tenocyte proliferation and/or viability may be susceptible to reactive oxygen species. For example, equine tenocytes show a decrease in proliferation when subjected to bolus addition of 10–100 μM of H_2_O_2_ (30). Also, recent studies show increased expression of peroxiredoxin 5 (PRDX5), a thioredoxin peroxidase with antioxidant properties, within tendinopathic tendons, suggesting that oxidative stress may be involved in the pathogenesis of tendinopathy (31).

Fibroblasts are able to generate ROS following a variety of biochemical and physical stimuli such as cytokines and growth factors (32–36). During cyclical loading of tendon, the period of maximum tensile load is associated with ischaemia. Subsequent restoration of normal tissue oxygenation may lead to enhanced ROS production (30). Exercising tendon core temperatures may reach 45°C (30), which may induce ROS production, most probably from the mitochondria (9).

A further, though highly speculative possibility, is that tendons are indirectly influenced by changes in ROS metabolism in other tissues and cells such as within exercising muscle (9, 37–39). In addition, although the extent of enhancement is contested (37), exhaustive exercise appears to increase ROS generation by activated phagocytes (37, 39–41). Phagocytic activity involves the generation and release, to the phagosome, of O_2^−^_, H_2_O_2_, HO, ^1^O_2_, HOCl and minor quantities of NO (42), an undesirable effect of which is the potential of collateral damage to ‘normal’ cells and tissues through extracellular bursts of ROS and ROS leakage (42–44). This change in granulocyte activity may also have more general consequences for ROS levels in tissues other than skeletal muscle, possibly including the tendon, through collateral exposure to ROS or mediators/signals arising from their actions. More specifically, while the underlying tendon degeneration does not appear to involve inflammation, micro-tears may be followed by local inflammatory reactions (12), which can disrupt tendon structure (8).

The potential nature of ROS involvement in tendinopathy or in post-rupture tendon healing is only speculative. Tenocyte proliferation, development and function may be susceptible to influence by endogenous or exogenous sources of ROS exposure.

The tendon matrix may be prone to direct or indirect modification by ROS. For example, increased O_2^.−^_ production in ischaemic rat skin is correlated with impairment of wound healing and antioxidant treated skin possessed greater amounts of organised collagen relative to ischaemic controls (45, 46). O_2^.−^_ stimulates collagen biosynthesis in rat dermal fibroblasts (47).

With regard to tenocyte proliferation, from studies on avian tenocytes, mechanical load and growth factors (e.g. platelet derived growth factor [PDGF] and insulin-like growth factor-I [IGF-I]) may work in concert to stimulate tenocyte cell division during healing (48). These growth factors influence DNA synthesis and cell division in a potentially redox-sensitive manner. For example, PDGF stimulation of rat vascular smooth muscle cells transiently increases intracellular H_2_O_2_ concentration, and H_2_O_2_ is required for PDGF signal transduction (49). In addition, proliferation and migration of vascular smooth muscle cells is inhibited by the H_2_O_2_ scavenger, catalase (50).

Extracorporeal shock wave therapy (ESWT) promotes tendon repair and bone growth (51), induces elevated O_2^.−^_ production which mediates extracellular signal regulated kinase signal transduction during osteogenesis (52). Differentiation was not influenced by inhibition of H_2_O_2_, peroxynitrite or nitric oxide production, suggesting a specific involvement of O_2^−^_. Whether such observations are relevant to the effects of ESWT on tenocytes and tendon repair remains to be determined.

Tenocyte viability may be compromised by ROS which are intimately associated with cell damage. Cell death is most often discussed in terms of ‘apoptosis’ and ‘oncosis’ (also referred to as necrosis). Apoptosis is an ordered, highly regulated form of cellular suicide, and is induced during cell, tissue and organ development and homeostasis. Both premature induction and inhibition of apoptosis are important in various aspects of pathology. Apoptosis is induced via activation of either the mitochondrial or death receptor pathways, but it is characterised by cell shrinkage, chromatin condensation and marginalization at the nuclear envelope, DNA cleavage and the formation of membrane bound nuclear and cytosolic remnants, termed ‘apoptotic bodies’. Apoptotic bodies are normally phagocytosed before membrane damage occurs. Inflammation is normally avoided. In comparison, oncosis involves cell swelling, rapid membrane damage, leakage of cellular constituents and the provocation of inflammation. Both oncosis and apoptosis, apparently unrelated manifestations of cell death, may share certain common regulatory components, with the rapid loss of ATP levels being a characteristic feature of oncosis as opposed to apoptosis (53).

The relationship between cell death mechanism and ROS is complex (27). Bursts of ROS (27, 54) and reductions in antioxidant enzyme activity (55) frequently accompany the induction of apoptosis, and oxidative stress is often reported in the later phase of cell demise (27). However, high concentrations of hydrogen peroxide, and other ROs, can prevent apoptosis or induce oncotic cell death.

Evidence for the involvement of apoptosis in tendon pathology is gradually emerging. Degenerative joint disease of the knee, an age-related condition, is associated with higher susceptibility of periarticular tenocytes to Fas ligand induced apoptosis. These changes may contribute to decreased cellularity in degenerative tendons and promote their rupturing (56, 57). Apoptosis has also recently been detected in human tendinopathic tendons (58, 59), and the increased number of apoptotic tendon cells in affected tendon tissue could affect the rate of collagen synthesis and repair (8, 60). Oxidative stress-induced apoptosis in human tendon fibroblasts may be mediated via pathway(s) involving release of cytochrome c from mitochondria to the cytosol and activation of the protease caspase-3 (61, 62).

### Non-immortalized human tenocytes in culture

As a component of studies into the involvement of ROS in tenocyte behaviour, in particular in orchestrating proliferation and tendon wound responses, we have prepared human non-immortalized tenocyte cultures from normal and ruptured Achilles tendons (63).

Although primary or early passage cells will logically offer the greatest approximation to the *in situ* cell, it may be desirable to define a range of passages for which there is minimal or no phenotypic drift. This may allow greater flexibility in the number of experiments that may be performed from a single tissue source. Furthermore, components defining phenotypic drift will be markers for altered cellular function and development in response to wounding and oxidative stress.

Cell proliferation rate, morphology and collagen, integrin and decorin expression have been used as markers of phenotype and changes in these would indicate ‘drift’. Type I collagen is the main collagen in tendons. Approximately 95% of collagen in normal tendons is type I (1), with type III and V collagen present in smaller amounts. Tenocytes produce both fibrillar and non-fibrillar components of the extracellular matrix. In addition to collagen, a number of non-collagenous protein and proteoglycans (PGs) are present, which interact with the fibrillar collagen region network (2–5). The predominant PG in tendon is the small, leucine-rich, decorin (6). Decorin belongs to a family of structurally related extracellular matrix PGs and glycoproteins. Integrins are a family of cell membrane glycoproteins. They mainly mediate cell-extracellular matrix adhesion, and are also involved in cell-cell adhesion (7, 64, 65). Many integrins serve as cell membrane receptors. Extra-cellular ligands include fibronectin, laminin, and various collagens. The cytoplasmic domains of the receptor form connections with the cytoskeleton, so integrins serve as a link between the cytoskeleton and the extra-cellular matrix. In addition, we have sought to monitor telomere length, apoptosis and intracellular ROS levels as indicators of changing cell ‘stress’ status. Such markers may subsequently be utilised to monitor the influence of ROS on tenocytes.

Tenocytes have been successfully cultured from various species including chicken, dog, rabbit, rat, horse and humans (66). There are differences in the extent to which drift is encountered in cells from differing species. The pattern of collagen synthesis may be a sensitive indicator of ‘drift’. Chick embryo tendons contain predominantly type I collagen, but, although type I collagen production remained constant, tenocytes produce type III collagen in about 10% of cells within three days of culture with the level of production increasing with passage (67). Avian tendon cells lost their ability to synthesize large amounts of collagen in vitro culture compared with other cell proteins (61). In juvenile rabbit tenocytes, a decrease in type I collagen transcript levels occurred following passage from primary culture (68). Variations in the level of decorin transcripts was also observed in cultured rabbit tenocytes, and dedifferentiation of the tenocytes occurred in early passages.

This would suggest that cells only up to passage two could be an acceptable representation of the *in situ* tenocyte.

**Figure 1:**
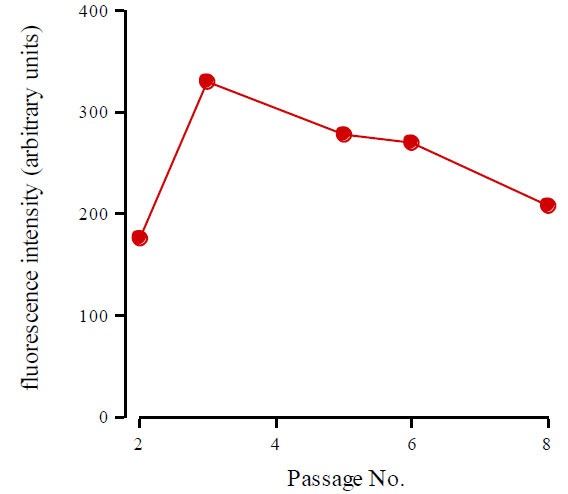
Reactive oxygen levels in tenocyte cultures measured following passage. ROS were detected via flow cytometric assessment of the median rhodamine fluorescence intensity following dihydrorhodamine loading

However, it is more than conceivable that drift will differ with the species, type and pathological state of the tendon. We have routinely cultured tenocytes from ruptured and non-ruptured Achilles human tendon up to passage 9 (Figure 1). Using ‘Western immunoblotting’ and immuno flow cytometry, we have quantified the level of decorin, collagen I and III and the β_1_ component of the integrin receptor levels, and examining their distribution via immunohistochemistry of proliferating, confluent and post-confluent cultures (63). Cell morphology changed with increasing passage number; cells became more rounded, were more widely spaced at confluence and confluent cell density declined (*P*=0.009). We saw no change in total cell layer collagen content, but the ratio of type III to type I collagen increased from 0.60 at passage 1 to 0.89 at passage 8 (*P*<0.001). Decorin expression significantly decreased with passage number (*P*<0.001). Integrin expression did not change. This study showed that the phenotype of human tenocytes in culture rapidly drifts with progressive passaging. Consequently, we recommend using only 1^st^ and 2^nd^ passage cells to maintain a phenotype as close as possible to that pertaining *in vivo* (63).

We have been concerned that non-immortalized cultures might show progressive increased stress susceptibility with progressive passage. However, from passage 0 to 9 there is ‘harmonisation’ in the level of ROS production and no evidence of heightened ‘oxidative stress’ and/or loss of viability (63).

## Conclusion

Tendinopathies have a complex aetiology, and the precise characterisation of a role for ROS in aspects of tendon biology will be less than trivial, especially when considering the prevalence of such ailments in sport and exercise medicine (69–80). However, reactive oxygen species may well be involved in tendon pathology and tendon healing (17). Characterised non-transformed human tenocyte cultures offer the potential to dissect tendon specific responses to reactive oxygen and oxidative stress.
